# Differential Regulation of CD4+ T Cell Adhesion to Cerebral Microvascular Endothelium by the β-Chemokines CCL2 and CCL3

**DOI:** 10.3390/ijms131216119

**Published:** 2012-11-30

**Authors:** Kenneth KY Liu, Katerina Dorovini-Zis

**Affiliations:** 1 Department of Pathology and Laboratory Medicine, The University of British Columbia, Vancouver, BC V5Z-1M9, Canada; E-Mail: kenliu@alumni.ubc.ca; 2 Division of Neuropathology, Department of Pathology and Laboratory Medicine, The University of British Columbia, Vancouver General Hospital, 855 West 12th Avenue, Vancouver, BC V5Z 1M9, Canada

**Keywords:** CD4+ T cell adhesion, CCL2, CCL3, blood-brain barrier

## Abstract

In Multiple sclerosis (MS), circulating lymphocytes cross the blood–brain barrier (BBB) and accumulate at sites of antigenic challenge. This process depends on specific interactions between lymphocytes and cerebral microvascular endothelium that involve endothelial activation by cytokines and the presence of chemokines. Chemokines play a key role in the orchestration of immune responses, acting both as chemoattractants and activators of leukocyte subsets. In the present study, we investigated the effects of the β-chemokines, CCL2 and CCL3, on the adhesion of CD4+ T cell subsets to human brain microvessel endothelial cells (HBMEC). Chemokines added to the lower compartment of a two-chamber chemotaxis system under confluent resting or cytokine-activated HBMEC, diffused through the culture substrate and bound to the basal surface of HBMEC. The low rate of adhesion of naïve, resting and memory CD4+ T cells to resting HBMEC was significantly upregulated following treatment of HBMEC with TNF-α and IFN-γ. Recently activated CD4+ T cells readily adhered to resting monolayers. Concentration gradients of CCL2 upregulated the adhesion of activated CD4+ T cells to cytokine treated but not resting HBMEC. The presence of CCL3 in the lower chamber increased the adhesion of memory T cells to both unstimulated and cytokine-treated HBMEC. These findings emphasize the importance of brain endothelial cell activation and the role of CCL2 and CCL3 in regulating the adhesion of CD4+ T cell subsets to BBB endothelium, thus contributing to the specificity of immune responses in MS.

## 1. Introduction

Multiple sclerosis (MS) is an autoimmune inflammatory disease of the central nervous system (CNS) characterized pathologically by discrete areas of inflammation, demyelination and variable axonal damage. Central to the initiation and evolution of the disease is the recruitment of lymphocytes and monocytes to the CNS, presumably in response to the local presence of a target antigen. The migration of effector cells to areas of the CNS that contain their cognate antigen takes place across the blood–brain barrier (BBB) that normally restricts their entry into the CNS. The BBB is a dynamic barrier formed by specialized endothelial cells lining the cerebral, spinal cord and pial microvessels. Under normal conditions, highly impermeable interendothelial tight junctions and absence of a vesicular transport system constitute the main barrier to the transport of proteins and lipid insoluble substances and the passage of hematogenous cells from blood to brain. Under inflammatory conditions, signaling events induced by proinflammatory cytokines lead to profound molecular, functional and morphological alterations of cerebral endothelial cells. Prominent among these changes is the *de novo* expression and upregulation of endothelial cell adhesion molecules and chemokines which, through specific interactions with corresponding ligands on leukocytes, provide the necessary cues for their adhesion and migration across the BBB.

Chemokines are a family of small (8- to 20-kDa) secreted chemoattractant cytokines that have been associated with several biological and pathological processes in the CNS, including a central regulatory role in neuroinflammation. Chemokines are classified into four groups based on the number and spacing of the conserved cysteine residues: CXC (α), CC (β), CX3C (δ) and XC (γ) [1,2]. The β-chemokines CCL2 (MCP-1) and CCL3 (MIP-1α) have been increasingly implicated in CNS inflammation. CCL2 was the first CC chemokine to be characterized biologically and shown to attract monocytes, but not neutrophils [3]. It binds the receptor CCR2 with high affinity and chemoattracts CCR2+ leukocytes such as monocytes, memory T lymphocytes and natural killer cells. In humans, CCR2 is expressed by virtually all monocytes and approximately 15% of CD4+ T cells in the circulation that also express markers of chronic activation such as CD26 [4]. CCL3 binds the receptors CCR1 and CCR5 on leukocytes and is a potent chemoattractant for monocytes and immature dendritic cells [5]. Both chemokines have been localized on astrocytes, macrophages and microglia in the center of acute demyelinating plaques and in the adjacent white matter [6]. Expression of CCL2 has been documented on reactive astrocytes and inflammatory cells in acute and chronic active MS lesions [7,8]. The levels of CCL2 in the cerebrospinal fluid have been reported low in active MS possibly because of continuous binding and internalization of CCL2 by CCR2 expressing monocytes transmigrating across the BBB [9]. Following treatment with interleukin-1β (IL-1β), astrocytes released increased levels of CCL3 protein [5,10]. Although the expression of chemokines by glial cells has been relatively well documented [11,12], the expression of chemokines by human brain microvessel endothelial cells (HBMEC) has not been fully characterized. Previous studies from our laboratory investigated the kinetics of expression and cytokine-induced upregulation and release of CCL2 and CCL3 by HBMEC *in vitro* and showed that under unstimulated conditions HBMEC constitutively synthesize and release low levels of CCL2, whereas CCL3 is minimally expressed and not released. Incubation with tumor necrosis factor-α (TNF-α), IL-1β, or bacterial lipopolysaccharide (LPS) significantly upregulated the expression and release of both chemokines in a time-dependent manner [13].

T lymphocytes of the CD4+ and CD8+ phenotype participate in the perivascular cuffs within demyelinated MS plaques and their numbers increase in parallel with the maturation of the lesions, indicating active continuous recruitment across the BBB [14,15]. Although CCL2, CCL3 and their receptors have been previously detected in inflammatory and glial cells in MS lesions, their role in the chemoattraction of T cell subsets to sites of antigenic challenge in the brain has not been previously addressed. In the present study, we investigated the effects of CCL2 and CCL3 on the adhesion of CD4+ T cells to human cerebral endothelial cells using a well-characterized *in vitro* model of the BBB. We demonstrate that the presence of CCL2 or CCL3 concentration gradients across the monolayers differentially regulates T cell adhesion and this effect is dependent upon the subset of T cells and the activation state of the BBB endothelium. These findings lend further support to the specificity of chemokine signaling during lymphocyte transmigration across the BBB and the importance of the cerebral endothelium in determining the composition of the inflammatory infiltrates in the CNS.

## 2. Results and Discussion

### 2.1. Chemokine Diffusion Across HBMEC Monolayers

**Figure 1:**
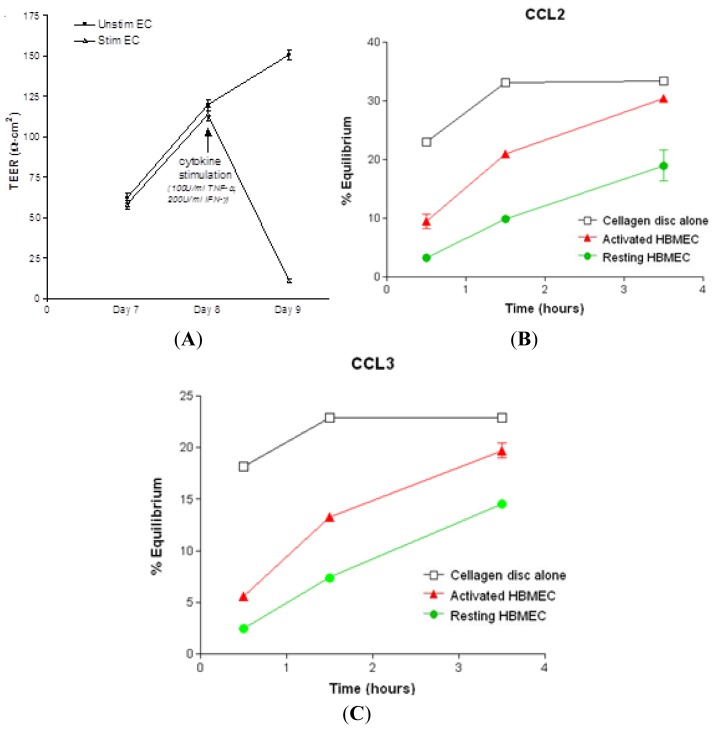
Diffusion of ^125^I-labelled chemokines across resting and cytokine treated HBMEC monolayers. (**A**) Confluent HBMEC monolayers grown on Cellagen discs were used when the TEER reached over 100 Ω cm^2^ by day 8 after plating. Stimulation of HBMEC with 100 U/mL TNF-α and 200 U/mL IFN-γ for 24 h resulted in drastic decrease of TEER to less than 25 Ω cm^2^. Radiolabeled CCL2 (**B**) and CCL3 (**C**) readily diffuse across the Cellagen membrane from the lower to the upper chamber in the absence of endothelial monolayers. The diffusion of labeled chemokines across unstimulated HBMEC is slow. The rate of chemokine diffusion across HBMEC was increased following 24 h incubation with 100 U/mL TNF-α and 200 U/mL IFN-γ and corresponded with the decrease of TEER across the monolayers. The data shown are representative of two experiments.

The rate of diffusion of CCL2 and CCL3 across confluent HBMEC monolayers in the presence of chemokine gradients was determined in a double-chamber chemotaxis system using radiolabeled chemokines. Confluent resting monolayers with transendothelial electrical resistance (TEER) greater than 100 Ω cm^2^ restricted the passage of CCL2 and CCL3 to the upper chamber, as compared to Cellagen^®^ discs alone during the entire 3.5 h of the assay (Figure 1B,C). Treatment of confluent HBMEC monolayers with 100 U/mL TNF-α and 200 U/mL interferon-γ (IFN-γ) for 24 h decreased the TEER to less than 25 Ω cm^2^ (Figure 1A) and led to a rapid increase of chemokine diffusion at 1.5 and 3.5 h, compared to resting HBMEC (Figure 1B,C). In the absence of HBMEC monolayers, there was a steep increase in percent equilibrium between 0.5 and 1.5 h. The rate of equilibrium for CCL2 and CCL3 reached a plateau after 1.5 h.

These results indicate that unstimulated HBMEC monolayers delay the basolateral-to-apical passage of CCL2 and CCL3, whereas diffusion through cytokine-pretreated cultures occurs rapidly at different rates and is directly related to the increased permeability of the monolayers. This pattern of diffusion is similar to that previously reported for the β-chemokines CCL4 and CCL5 and the chemokine CXCL12 across confluent resting and cytokine stimulated HBMEC monolayers [16,17]. It is conceivable that chemokine diffusion across cytokine-treated cultures follows a paracellular route, since it coincided with a significant increase in the permeability of the monolayers, as indicated by the decrease in TEER. This is consistent with previous observations showing that treatment of HBMEC with TNF-α or IFN-γ increases the permeability of the cerebral endothelial tight junctions to macromolecules [18,19]. Both TNF-α and IFN-γ have been identified on perivascular inflammatory cells, macrophages and astrocytes in acute and chronic active MS lesions [20–23]. This suggests that upon local synthesis and release into the perivascular space, these cytokines act directly on the cerebral microvasculature to increase the permeability of the BBB and facilitate the diffusion of chemokines produced by resident CNS cells during the inflammatory response. In an *in vitro* mouse model of the BBB, exposure of resting cultures to CCL2 reduced the level of expression and altered the distribution of zonula occludens (ZO-1) and occludin at the interendothelial tight junctions, a mechanism that could make abluminal chemokines available to infiltrating leukocytes [24], although the effect of these changes on the permeability of the tight junctions was not assessed. In the present study, incubation of HBMEC with CCL2 or CCL3 for 30 min did not alter the TEER across HBMEC monolayers (data not shown) suggesting that neither chemokine had any effect on junctional permeability. These findings are consistent with a previous report from our laboratory indicating that incubation with two other β-chemokines, CCL4 or CCL5, did not alter the permeability of the HBMEC monolayers over a 24-h period [16]. It is now well accepted that changes in the expression or distribution of junctional proteins do not necessarily correspond to changes in junctional permeability. A recent study reports no changes in the permeability of human brain endothelial cells after treatment with 200 or 500 ng/mL CCL2 for 30–45 min, even though CCL2 induced transient disruption of the adherens junctions [25]. Previous studies have shown that CCL2 can be transported transcellularly from the abluminal to the luminal side via binding to its receptor, CCR2, and subsequently carried to caveolar vesicles for transcytosis [26]. Such a mechanism could be implicated in the minimal and much slower transport of the chemokines across unstimulated cultures with high TEER, but not in the rapid diffusion across cytokine-stimulated cultures.

### 2.2. Localization and Binding of CCL2 and CCL3 to HBMEC

**Figure 2:**
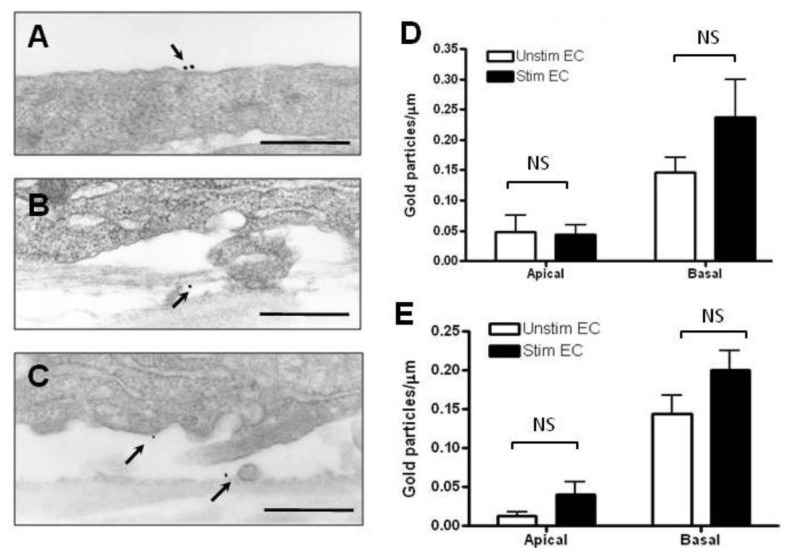
Immunoelectron microscopic localization of CCL2 (**A**, **B**, **D**) and CCL3 (**C**, **E**) on HBMEC 3 h after the addition of 500 ng/mL CCL2 or 100 ng/mL CCL3 to the lower chamber. The number of gold particles bound to the apical (**A**) and basal (**B**) surface (arrows) of either resting or cytokine-stimulated HBMEC is similar. (**D**) Quantification of the apical and basal binding of CCL2 in unstimulated (open bars) and stimulated (solid bars) HBMEC. The number of gold particles counted was 55 for the resting and 78 for the cytokine treated cultures. (**C**) Gold particles (arrows) indicate binding of CCL3 to the basolateral cell membrane and the subendothelial basal lamina-like material of cytokine-treated HBMEC. (**E**) Quantification of the apical and basal binding of CCL3 in unstimulated (open bars) and stimulated (solid bars) HBMEC. The number of gold particles counted was 63 for the resting and 82 for the cytokine treated cultures. There is a slight but not statistically significant increase in basal binding of CCL2 or CCL3 after TNF-α and IFN-γ stimulation. A slight increase of apical CCL3 but not CCL2 binding was observed after TNF-α and IFN-γ stimulation. The data shown are from one experiment for each chemokine. Scale bars = 0.5 μm.

**Figure 3:**
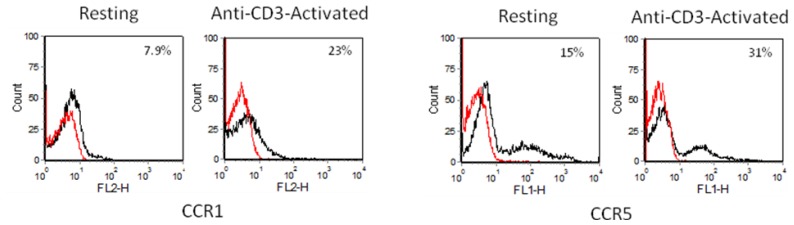

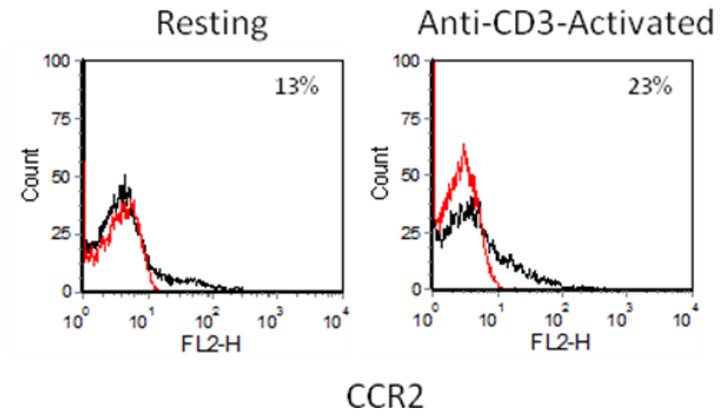
Chemokine receptor expression on resting and anti-CD3-activated CD4+ T cells as determined by flow cytometry. The black lines indicate the chemokine receptor tested and the red lines represent isotype controls. The percentage of cells expressing each chemokine receptor is shown at the top right hand corner. All 3 chemokine receptors are upregulated in anti-CD3-activated CD4+ T cells. The data shown are from one of three independent experiments.

Immunoelectron microcopy was performed to localize and quantify the binding of CCL2, and CCL3 to the apical and basal endothelial surfaces and the subendothelial matrix. CCL2 bound mostly to the basal cell surface and the subendothelial matrix of both unstimulated and cytokine stimulated HBMEC (Figure 2B). Very few gold particles were found on the apical surface (Figure 2A). There was a modest, but not significant increase in the number of gold particles bound to the basal surface after cytokine stimulation of HBMEC (Figure 2C), whereas the apical density remained unchanged. Similarly, the binding of CCL3 was mostly basal under both stimulated and unstimulated conditions (Figure 3A). However, there was a trend towards increase in both apical and basal binding following cytokine stimulation (Figure 3B), but this did not reach statistical significance. Thus, the binding of CCL2 and CCL3 to the endothelium and the extracellular matrix in the presence of chemokine gradients was not significantly different between the two chemokines under resting and cytokine-stimulated conditions.

The distribution of chemokines along the apical (luminal) and basal (abluminal) surface of the endothelium is of physiological significance, since chemokines bound to the luminal surface trigger integrin activation and firm adhesion, whereas chemokines distributed along the basal surface and subendothelial matrix establish the haptotactic gradients necessary for transendothelial migration [27]. The binding patterns of CCL2 and CCL3 are predominately basolateral in resting and cytokine-treated HBMEC. It is possible that some of the apically bound chemokines represent endogenously synthesized and membrane-bound CCL2 and CCL3, since both chemokines are produced and released by activated HBMEC [13]. The predominantly basolateral pattern of distribution of “abluminally” added CCL2 and CCL3 is similar to that previously observed for the β-chemokines CCL4 and CCL5 placed under confluent HBMEC monolayers, although binding to the basal surface was significantly greater as compared to the apical cell membrane [16]. In the presence of chemokine gradients, the α-chemokine, CXCL12, exhibits a polarized distribution in favor of the basolateral surface in resting HBMEC followed by redistribution to the apical surface upon cytokine stimulation, resulting in equal binding along the basal and apical surfaces [17]. In this respect it is interesting that the binding of chemokines to endothelial cells derived from different organs can vary greatly [28]. The differential binding of various chemokines to resting and cytokine-activated HBMEC may well reflect changes in the surface charge of endothelial cells and the chemical structure and distribution of glycosaminoglycans in the course of the inflammatory response [29]. TNF-α and IFN-γ have been shown to induce redistribution of endothelial surface anionic sites and alter the metabolism of glycosaminoglycans [30]. Thus, the changes in the distribution of CCL2 and CCL3 on the endothelial surface in the present study are likely the result of changes in the distribution and/or chemical structure of the heparan-sulfate glycosaminoglycan chains following cytokine treatment. Moreover, the differential presentation along the apical and basolateral endothelial surfaces is likely of functional significance in the recruitment of lymphocyte subsets across the endothelial barrier.

### 2.3. Chemokine Receptor Expression on Leukocyte Subsets

Chemokines exert their actions by signaling through their G-protein coupled, pertussis toxin-sensitive seven-transmembrane receptors on leukocytes. Each chemokine receptor can bind multiple chemokines and most chemokines bind more than one receptor. CCL2 binds the receptor CCR2, whereas CCL3 signals through two receptors, CCR1 and CCR5. The level of expression of these receptors was assessed by flow cytometry on four CD4+ T cell subsets.

#### 2.3.1. Resting and Activated CD4+ T Cells

Up to 15% of resting CD4+ cells expressed the receptors CCR1, CCR2 and CCR5. Anti-CD3 activation of CD4+ T cells for 24 h increased the surface expression of all three receptors. Both resting and activated CD4+ T cells stained brightly for CCR5 as compared to CCR1 and CCR2 (Figure 3).

#### 2.3.2. CD4+CD45RO-(Naïve) and CD4+CD45RA- (Memory) T Cells

**Figure 4:**
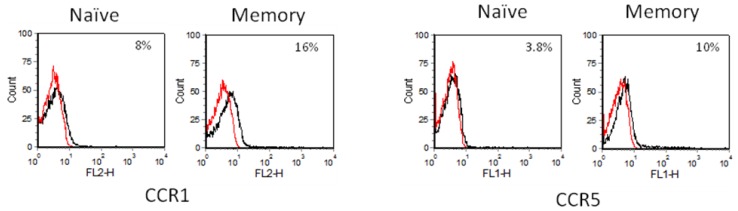

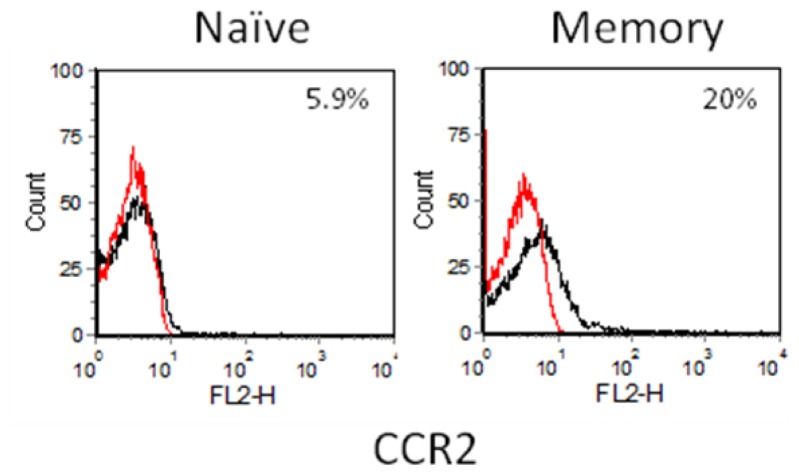
Chemokine receptor expression on CD45RA-(memory) and CD45RO-(naïve) CD4+ T cells as determined by flow cytometry. The black lines indicate the chemokine receptor tested, and the red lines represent isotype controls. The percentage of cells expressing each chemokine receptor is shown at the top right hand corner. Naïve CD4+ T cells display minimal expression of CCR1, 2 and 5. All three chemokine receptors are upregulated in memory CD4+ T cells. The data shown are from one of three independent experiments.

**Figure 5:**
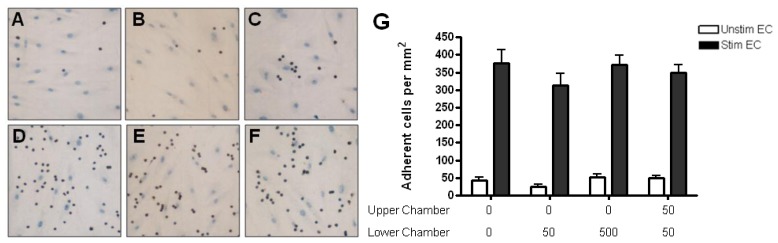
Adhesion of resting CD4+ T cells to HBMEC in response to CCL2. (**A**) A few resting T cells (brown) adhere to unstimulated HBMEC (light blue). (**D**) Cytokine treatment significantly increases the number of adherent T cells. The presence of CCL2 in the subendothelial region has no effect on binding to unstimulated (**B**) or cytokine-treated (**E**) HBMEC. The presence of CCL2 in both chambers (no gradients) has no effect on T cell adhesion to resting (**C**) or cytokine-treated (**F**) HBMEC. (**G**) Quantification of resting CD4+ T cell adhesion to resting and cytokine-activated HBMEC. Values represent mean ± SEM of duplicate wells from 3 experiments.

Further separation of the resting CD4+ T cell population into naïve and memory phenotypes based on their different CD45 isoforms showed that memory T cells are the main cell type expressing CCR1, CCR2 and CCR5. Less than 10% of naïve CD4+ T cells expressed these three β-chemokine receptors (Figure 4). In contrast to the resting CD4+ T cell population, CCR5^bright^ cells were absent in both resting naïve and memory CD4+ pools (Figures 4 and 5). This suggests that CCR5^bright^ cells belong to the CD45RA+/45RO+ double positive CD4+ T cell subset, which was removed during the isolation of naïve or memory CD4+ T cells.

### 2.4. Adhesion of CD4+ T Cell Subsets to HBMEC in Response to CCL2 and CCL3

The pattern of chemokines and their receptors expressed at the inflammatory site reflect the range of inflammatory cells recruited to the site. CCR5, a receptor for CCL3, CCL4 and CCL5, is expressed preferentially on memory, recently activated T cells and Th1 clones [31,32] and has been localized on lymphocytes in actively demyelinating MS lesions [33]. CCR1 is preferentially expressed on CD4+ T cells with the CD45RO+ phenotype and has been localized on T cells and macrophages within active MS lesions [34]. In chronic MS lesions, CCR2 and CCR5 were present on infiltrating lymphocytes, macrophages and activated microglia, and their ligands, CCL2 and CCL3 co-localized around blood vessels with infiltrating lymphocytes [35].

**Figure 6:**
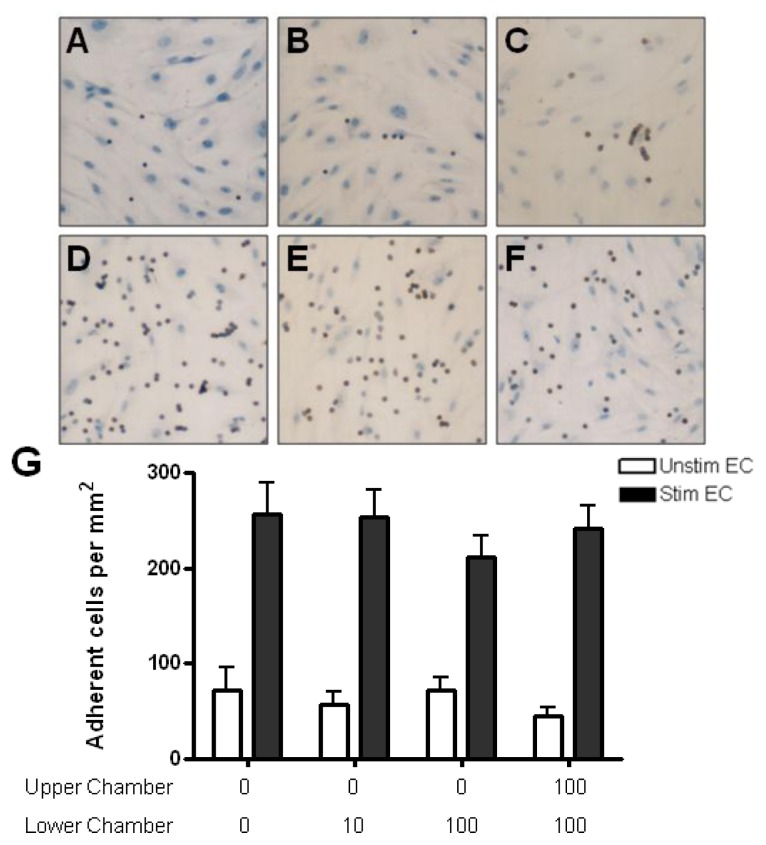
Adhesion of resting CD4+ T cells to HBMEC in response to CCL3. (**A**) A small number of CD4+ T cells adhere to resting HBMEC. (**D**) Cytokine treatment significantly increases the number of adherent T cells. The presence of CCL3 gradients has no effect on adhesion to unstimulated (**B**) or cytokine-treated (**E**) HBMEC. Similarly, the presence of CCL3 in both chambers has no effect on adhesion to unstimulated (**C**) or cytokine-treated HBMEC (**F**). (**G**) Quantification of resting CD4+ T cell adhesion to resting and cytokine-activated HBMEC. Values represent mean ± SEM of duplicate wells from three experiments.

Adhesion of resting CD4+ T cells to unstimulated HBMEC was low (~50 cells/mm^2^). Treatment of HBMEC with TNF-α and IFN-γ for 24 h induced a seven-fold increase in adhesion to HBMEC (up to 400 cells/mm^2^) (Figures 5 and 6). The presence of CCL2 or CCL3 gradients did not influence the adhesion to either resting or cytokine-stimulated HBMEC (Figures 5 and 6).

**Figure 7:**
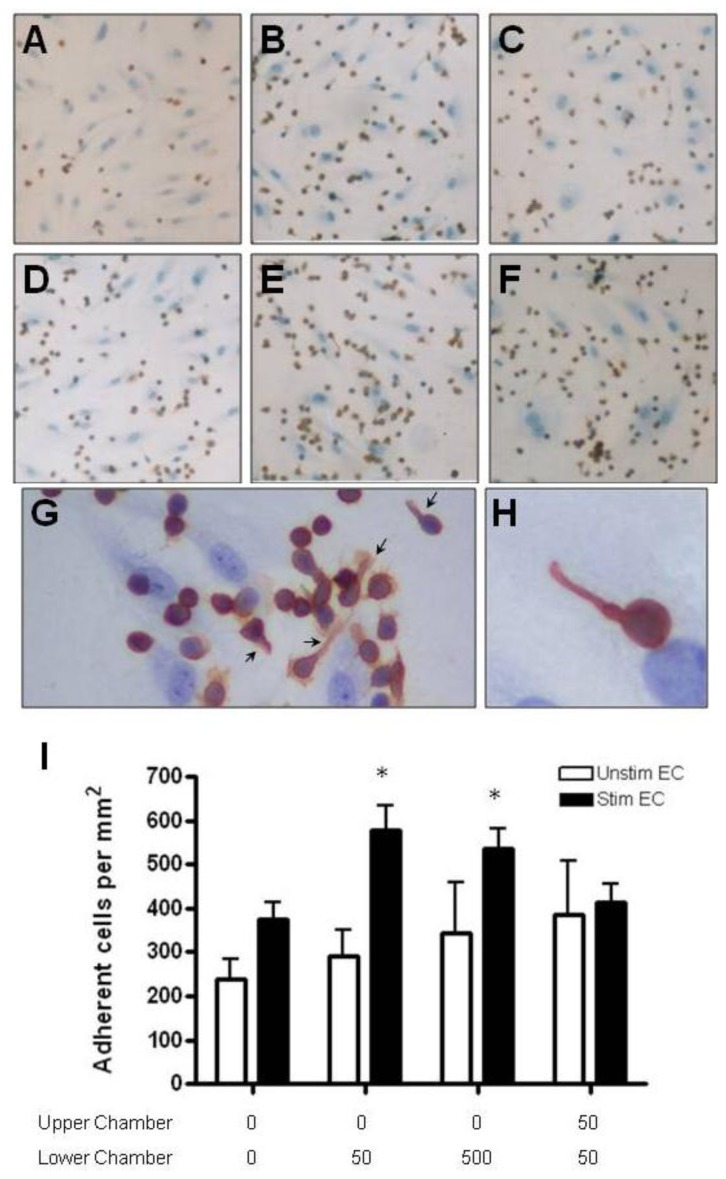
Adhesion of activated CD4+ T cells to HBMEC in response to CCL2. (**A**) Anti-CD3 activation of CD4+ T cells increases baseline adhesion to unstimulated HBMEC. (**D**) Stimulation of HBMEC with TNF-α and IFN-γ further increases T cell adhesion to the endothelium. CCL2 gradients (50 ng/mL) augment the adhesion of activated T cells to cytokine treated HBMEC (**E**) with only slight effect on binding to resting HBMEC (**B**). The presence of CCL2 in both chambers has no effect on adhesion to resting (**C**) or activated (**F**) HBMEC. In the presence of CCL2 in the lower chamber (500 ng/mL), several adherent T cells are polarized as they extend uropods in the opposite direction of the leading edge of the cell (**G** [arrows], **H**). Nonpolarized T cells often form clusters around cells with uropods (**G**). (**I**) Quantification of activated CD4+ T cell adhesion to resting and cytokine-activated HBMEC. CCL2 gradients in the lower chamber significantly increase the adhesion of activated CD4+ T cells to cytokine-activated HBMEC. Values represent mean ± SEM of duplicate wells from three experiments. * *p* < 0.05.

**Figure 8:**
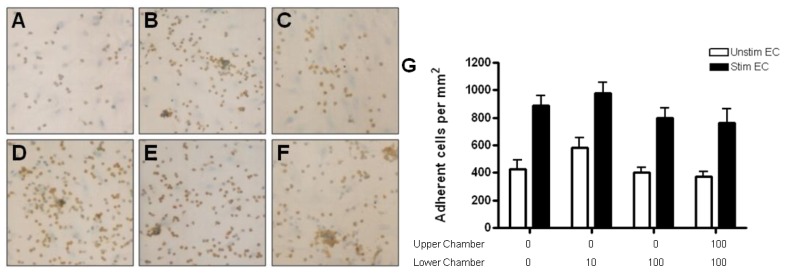
Adhesion of activated CD4+ T cells to resting and cytokine-treated HBMEC in response to CCL3. (**A**) Anti-CD3 activation of CD4+ T cells increases adhesion to unstimulated HBMEC. (**D**) Treatment of HBMEC with TNF-α and IFN-γ further increases T cell adhesion to the endothelium. The presence of CCL3 in the lower chamber (10 ng/mL) has no effect on binding to either resting (**B**) or cytokine treated (**E**) HBMEC. Adhesion of T cells to HBMEC is not affected when CCL3 is added to both chambers (**C**, **F**). Similar to CCL2, frequent clustering of adherent T cells occurs in the presence of CCL3 (**B**, **D**–**F**). (**G**) Quantification of resting CD4+ T cell adhesion to resting and cytokine-activated HBMEC. Values represent mean ± SEM of duplicate wells from three experiments.

Anti-CD3-activated CD4+ T cells adhered to unstimulated HBMEC more readily than resting CD4+ T cells (>200 cells/mm^2^) (Figures 7 and 8). Adhesion was further augmented by two fold when HBMEC were treated with cytokines. In the presence of 50 or 500 ng/mL CCL2 in the lower chamber, a significantly greater number of activated CD4+ T cells adhered to cytokine treated but not resting HBMEC (Figure 7). The addition of CCL2 to both upper and lower chambers (no gradients) had no effect on adhesion. In contrast, CCL3 gradients had no effect on activated CD4+ T cell adhesion to either resting or cytokine stimulated HBMEC (Figure 8). In the presence of chemokines in the lower chamber, activated T cells adhering to endothelial cells often form small clusters and extend a uropod away from the cell body of the adherent lymphocyte (Figures 7E–H and 8E,F).

**Figure 9:**
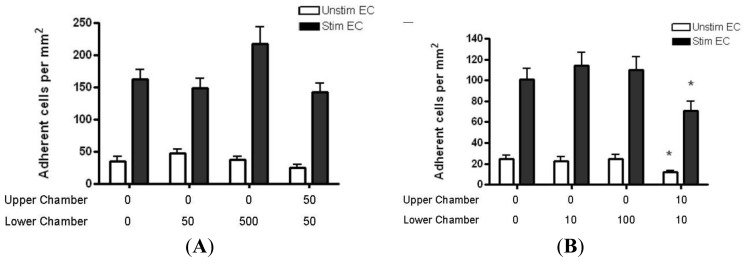
(**A**) Quantification of naïve CD4+ T cell adhesion to resting or cytokine-treated HBMEC in the presence of CCL2 (**A**) and CCL3 (**B**) gradients. Cytokine treatment upregulates adhesion; however, neither CCL2 nor CCL3 increased adhesion of naïve CD4+ T cells to resting or cytokine-treated HBMEC. Interestingly, there is a significant decrease in adhesion of naïve T cells when CCL3 is present in both chambers (**B**). Values represent mean ± SEM of duplicate wells from two experiments. *p < 0.05 as compared to controls.

Only a small number of naïve CD4+ T cells adhered to resting HBMEC monolayers (<50 cells/mm^2^). Cytokine stimulation of HBMEC increased naïve CD4+ T cell binding to HBMEC by two fold (>100 cells/mm^2^). The presence of CCL2 or CCL3 gradients did not further increase adhesion to unstimulated or cytokine stimulated HBMEC (Figure 9). Interestingly, the presence of CCL3 in both upper and lower chambers (absence of gradients) decreased the adhesion of naïve T cells to both unstimulated and stimulated monolayers (Figure 9B).

**Figure 10:**
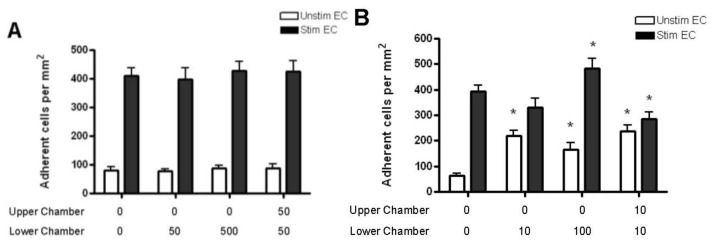
Quantification of memory CD4+ T cell adhesion to resting and cytokine-activated HBMEC in the presence of CCL2 (**A**) and CCL3 (**B**) gradients. CCL2 has no effect on the adhesion of memory CD4+ T cell to resting or cytokine-activated HBMEC (**A**). CCL3 added to the lower or both chambers increases memory T cell adhesion to resting HBMEC. The presence of 100 ng/mL of CCL3 in the lower chamber increases memory T cell adhesion to cytokine-activated HBMEC, whereas 10 ng/mL of CCL3 in both chambers decreases adhesion. Values represent mean ± SEM of duplicate wells from two experiments. *p < 0.05 as compared to controls.

Memory T cells adhered more readily than naïve T cells to both resting and stimulated HBMEC monolayers (>50 cells/mm^2^). The presence of CCL2 gradients had no effect on the adhesion of memory T cells (Figure 10A), whereas CCL3 increased their adhesion to unstimulated endothelium (Figure 10B). When memory T cells were incubated with stimulated HBMEC, adhesion was upregulated in the presence of CCL3 in the lower chamber at the highest concentration (100 ng/mL). The addition of 10 ng/mL of CCL3 to both chambers resulted in decreased adhesion (Figure 10B).

Chemokines play a central role in the recruitment of inflammatory cells to sites of antigenic challenge in the CNS. Chemokines are synthesized and released by inflammatory cells and resident CNS cells, such as astrocytes and microglia, in response to inflammatory stimuli, and are delivered to the extracellular space where, upon reaching the subendothelial region, they bind glycosaminoglycans along the abluminal surface of the endothelium and the basal lamina. Immobilization to glycosaminoglycans serves to protect chemokines from proteolytic cleavage and establish the necessary gradients for the directional migration of leukocytes. Chemokines synthesized and released by endothelial cells, as well as those diffusing across the endothelium from the abluminal side, bind to glycosaminoglycans on the luminal surface and provide signals for integrin activation and firm adhesion [16,17]. In the present study, the adhesion of resting CD4+ T cells to unstimulated HBMEC was minimal, reflecting the absence of non-activated T cells trafficking across the BBB under normal physiological conditions and the lack of or low constitutive expression of adhesion molecules by cerebral endothelial cells under resting conditions [36–39]. Following a 24-h treatment with TNF-α and IFN-γ, adhesion increased up to seven-fold, consistent with maximal upregulation of intercellular adhesion molecule-1 (ICAM-1) and vascular cell adhesion molecule-1 (VCAM-1) on HBMEC [36,37]. These observations are in agreement with our previous studies, showing that adhesion of resting T cells to HBMEC is dependent upon the activation status of cerebral endothelial cells and is mediated by ICAM-1-lymphocyte function-associated antigen-1 (LFA-1) and VCAM-1-very late antigen-4 (VLA-4) interactions [40]. Neither CCL2 nor CCL3 increased the adhesion of resting CD4+ T cells to unstimulated or cytokine-stimulated HBMEC.

In keeping with previous observations [16], the recently activated polyclonal population of CD4+ T cells readily adhered to resting endothelial cells with further increase in adhesion after cytokine stimulation of HBMEC. CCL2 augmented the adhesion of activated CD4+ T cells to cytokine-stimulated but not unstimulated HBMEC consistent with the higher expression of the CCR2 receptor on CD4+ T cells after anti-CD3 activation. In the presence of chemokines, activated T cells adhering to activated HBMEC often formed groups of closely associated cells. In addition, the adherent cells frequently assumed a polarized shape with a long cytoplasmic process (uropod) extending away from the leading adhering cell body. It has been previously shown that the chemokine-induced polarization of T cells requires actin polymerization in the leading edge and myosin-dependent contractility in the trailing uropod, which is orchestrated by the activation of small GTPases of the Ras and Rho family [41–43]. Previous studies indicate that ICAM-1 and ICAM-3 redistribute to the uropod and that signaling through the uropod enables polarized T cells to contact and recruit other T cells [44]. Based on these observations, it is likely that signaling of polarized activated CD4+ T cells through uropod formation in our study mediated the cell clumping in the presence of chemokines. A similar increase in adhesion of activated CD4+ T cells to cytokine-treated, but not resting HBMEC monolayers was previously observed in the presence of concentration gradients of the β-chemokines CCL4 and CCL5 [16]. CCL3 did not have any effect on the adhesion of activated CD4+ T cells regardless of the activation status of the endothelium and the increased expression of CCR1 and CCR5 on anti-CD3-activated T cells. Our results are consistent with a previous study reporting that CCL4 and CCL5, but not CCL3 increased the adhesion of anti-CD3-activated CD4+ T cells to umbilical vein endothelial cells stimulated with interleukin-1α (IL-1α) [45]. CCL3 is an important chemokine in the pathogenesis of neuroinflammation since the Th1-associated chemokines CXCL10, CCL3 and CCL5 were significantly elevated in cerebrospinal fluid samples collected from MS patients as compared to control groups [46]. CCL3 is a more potent T lymphocyte chemoattractant than CCL4 *in vitro* with a biphasic lymphocyte response at concentrations of 100 pg/mL and 10 ng/mL [47].

The maturation and differentiation of T cells correlates with the expression of different isoforms of the leukocyte common antigen (CD45). In the absence of chemokines, memory (CD4+ CD45RA−) T cells uniformly adhere and migrate more readily than naïve (CD4+CD45RO−) T cells across resting and cytokine-activated HBMEC monolayers. Similar to our findings, it has been previously reported that up to 97% of the migrated CD4+ T cell population expresses memory phenotype [48]. Furthermore, the activation marker CD69 is also enriched in the migrated population, suggesting that activated memory T cells, which express CD69, constitutively traffic across the BBB. While the presence of CCL2 and CCL3 gradients had no effect on naïve CD4+ T cell adhesion, the presence of CCL3 in both upper and lower chambers (no gradients) significantly decreased naïve CD4+ T cell adhesion to both unstimulated and cytokine-stimulated HBMEC. The hyporesponsiveness of naïve CD4+ T cells to CCL3 has been shown through the binding of CCR1 on naïve T cells [49]. In addition, the presence of a relatively high concentration of CCL3 in the upper chamber might prematurely trigger internalization of CCR5 upon ligand binding, thereby decreasing the ability of naive CD4+ T cell to firmly adhere due to lack of integrin activation [50].

The adhesion of memory T cells was augmented by at least four-fold after cytokine-activation of the endothelial monolayers. The presence of CCL3 in the subendothelial region increased the adhesion of memory CD4+ T cells to resting HBMEC. When higher concentrations of CCL3 (100 ng/mL) were added to the lower chamber, memory CD4+ T cell adhesion to cytokine-treated HBMEC was also upregulated. This is in agreement with the flow cytometry data showing that memory T cells express higher levels of the chemokine receptors CCR1 and CCR5 on their surface as compared to naïve T cells. Thus, adhesion of memory T cells to cerebral endothelial cells is differentially regulated by the β-chemokines, since CCL4 and CCL5 upregulate adhesion only to activated HBMEC [16], CCL3 to both resting and activated HBMEC, whereas CCL2 has no effect on adhesion. Previous studies have shown that CCL2, CCL3 and CCL5 attract memory T cells only [51]. Considering that the expression of CCR1, CCR2 and CCR5 on T cells is increased in the cerebrospinal fluid of MS patients [52] and that Th-1 polarized memory T cells are preferentially recruited to the CNS in MS [53], it is apparent that CCL3 is important in the adhesion of memory T cells to the BBB endothelium. This selective recruitment of memory T cells may be due in part to higher levels of expression or to a high affinity state of the α4 integrins expressed on memory as compared to naïve T cells [54].

The present study points toward distinct roles for CCL2 and CCL3 in the chemoattraction of specific CD4+ T cell subsets across the BBB. Participation of these chemokines in MS pathogenesis is supported by their documented expression by microglial cells, astrocytes and inflammatory cells in MS plaques. Binding of the released chemokines to the subendothelial region would set up the stage for the recruitment of T cell subsets across he BBB to MS lesions by establishing the required chemotactic and haptotactic gradients.

## 3. Experimental Section

### 3.1. Isolation and Culture of Human Brain Microvessel Endothelial Cells

Primary cultures of HBMEC were established from normal brains obtained at autopsy, as previously described [55]. The study has complied with all institutional policies and was approved by the ethics committees of the University of British Columbia and the Vancouver General Hospital. The endothelial origin of the cells was determined by their strong positive staining for Factor VIII-related antigen, binding of Ulex europeaus agglutinin-1 (UEA-1), uptake of acetylated low-density lipoprotein, and high alkaline phosphatase activity. The isolated clumps of HBMEC were plated onto fibronectin-coated 96-well plates (Corning Plastics, Corning, NY, USA) or Cellagen discs CD-24 (MP Biomedicals, Solon, OH, USA) and cultured in medium 199 (M199) (Invitrogen, Burlington, ON, Canada) supplemented with 10% horse plasma-derived serum (PDS) (Cocalico Biologicals, Reamstown, PA, USA), 2 mM l-glutamine, 100 U/mL penicillin, 100 μg/mL streptomycin, 0.25 μg/mL amphotericin B (all from Gibco, Burlington, ON, Canada), 20 μg/mL endothelial cell growth supplement and 100 μg/mL heparin (both from Sigma, St. Louis, MO, USA) at 37 °C in a humidified 5% CO_2_/95% air incubator. Culture media were changed every 2 days and HBMEC cultures were used upon reaching confluence, 8–10 days after plating.

### 3.2. Isolation and Characterization of T Cell Subsets

Peripheral blood mononuclear cells (PBMCs) were isolated from heparinized venous blood of healthy donors by centrifugation in Histopaque-1077 density gradients (density = 1.077 g/mL, Sigma, St. Louis, MI, USA) according to manufacturer’s instructions. Human CD4+, CD4+CD45RO− and CD4+CD45RA-T cells were isolated from other mononuclear cells by negative selection using T cell recovery columns (Cedarlane Laboratories, Burlington, ON, USA). The purity of the isolated T lymphocytes was greater than 90% as determined by flow cytometry using the following panel of antibodies: CD14 PE, CD45 FITC, CD4 PE, CD8 FITC, CD3 PerCP, CD19 APC, CD3 FITC, CD16/56 PE, CD20 PE (all from Becton Dickinson, San Jose, CA, USA) on FACS Calibur using CELLQuest Pro software (version 4.0.2; Becton Dickinson, Mississauga, ON, Canada). Viability was greater than 97% by the trypan blue exclusion test.

### 3.3. Antibodies, Cytokines and Chemokines

Mouse anti-human leukocyte common antigen (LCA/CD45) (DAKO, Glostrup, Denmark) and horseradish peroxidase (HRP)-conjugated goat anti-mouse IgG (Jackson ImmunoResearch, West Grove, PA, USA) were used for staining adherent lymphocytes in adhesion experiments.

To characterize chemokine receptor expression on lymphocyte subsets, Phycoerythrin (PE)-conjugated anti-CCR1 (FAB145P) and anti-CCR2 (FAB151P) and Fluorescein (FITC)-conjugated anti-CCR5 (FAB181F) were used (all from R & D systems). Secondary PE-conjugated Goat F(ab’)2 anti-mouse IgG was purchased from Cedarlane Laboratories.

Human CCL2 and CCL3 were a kind gift from Dr. Ian Clark-Lewis, UBC Biotechnology Laboratory. Recombinant human TNF-α was obtained from Sigma (St. Louis, MO, USA). Recombinant human IFN-γ was obtained from the NIH AIDS Research and Reference Reagent Program.

### 3.4. CD4+ T Cell Activation

T cell activation was carried out by incubating CD4+ T cells for 24 h in 4-well plates precoated with mouse anti-human CD3 mAb (clone HIT3a, BD Pharmingen, San Diego, CA, USA) at 10 μg/mL in 10% AB serum in RPMI supplemented with 30 U/mL IL-2 (NIH AIDS Research and Reference Reagent Program). Flow cytometric analysis showed greater than 60% and 45% of the activated CD4+ T cells expressing CD25 and CD69, respectively, as compared to less than 25% and 1% of resting CD4+ T cells.

### 3.5. Chemokine Diffusion Assay

Chemokine diffusion assays were performed as previously described [16]. HBMEC were grown to confluence on permeable collagen membranes (Cellagen discs) mounted on plastic supports and placed inside individual wells of 4 or 24-well plates. This double chamber system allows the diffusion of small molecular weight substances across the collagen membrane from the basal-to-apical or apical-to-basal direction. Confluent monolayers with a transendothelial electrical resistance (TEER) greater than 100 Ω·cm^2^ were used untreated or following coincubation with TNF-α (100 U/mL) and IFN-γ (200 U/mL). HBMEC cultures grown in 100 μL of 10% PDS in M199 in the upper chamber were placed over 350 μL of 10% horse serum in M199 in the lower chamber of a 24-well plate containing ^125^I-labelled CCL3 (100 ng/mL) or CCL2 (500 ng/mL) and incubated for up to 3.5 h at 37 °C. Radioactivity in the supernatants from the upper and lower chambers was measured at 0.5, 1.5 and 3.5 h using a Beckmann Gamma 5500. Results were expressed as percent equilibrium, *i.e.*, the concentration of radioactive counts (cpm/μL) in the upper chamber divided by the concentration of radioactive counts in the lower chamber. Each experiment was performed twice using duplicate wells to test for chemokine diffusion. Controls consisted of chemokine diffusion across Cellagen discs in the absence of EC.

### 3.6. Immunoelectron Microscopic Localization of Chemokines

Surface localization of CCL2 and CCL3 following establishment of chemokine gradients across HBMEC was carried out by immunoelectron microscopy. CCL3 (100 ng/mL) or CCL2 (500 ng/mL) were added to the lower chamber of a 4-well plate and allowed to diffuse for 3 h across confluent resting or cytokine-treated (TNF-α and IFN-γ) HBMEC monolayers grown on Cellagen discs. The monolayers were then washed with wash buffer (1% BSA and 1% NGS in PBS) and incubated with 15 μg/mL of primary Ab (mouse anti-human CCL2 or CCL3) for 40 min. Secondary goat anti-mouse IgG Ab conjugated to 10 nm gold particles was added for 60 min at room temperature. Cells were then washed and fixed in 1/2 strength Karnovsky’s fixative (2.5% glutaraldehyde and 2% PFA) in 0.2 M Cacodylate buffer for 1 h at 4 °C, washed with cacodylate buffer, post-fixed in 1% OsO_4_ for 1 h at 4 °C, washed with cacodylate buffer and block stained overnight in uranyl Mg acetate. Following dehydration in a graded series of methanol, the cultures were embedded in Epon-Araldite. Thin cross sections were viewed under a Zeiss 10 electron microscope following lead citrate staining. Quantification of the number of gold particles associated with the apical, basolateral cell membranes and the subendothelial basal lamina-like material was carried out by photographing one hundred cells from each resting or cytokine treated cultures at 25,000× magnification and counting the number of gold particles bound per micrometer of cell membrane, taking into account the magnification.

### 3.7. T Cell Adhesion Assay

Confluent HBMEC monolayers grown on Cellagen discs in duplicate wells were used unstimulated or stimulated with TNF-α (100 U/mL) and IFN-γ (200 U/mL) for 24 h to upregulate endothelial cell adhesion molecules and to mimic inflammatory conditions. The HBMEC monolayers were washed twice with 10% horse serum in M199 and chemokine gradients were established by adding chemically synthesized CCL2 or CCL3 in the lower chamber for 30 min to allow diffusion of the chemokines through the Cellagen membrane and binding to the EC. As a result, both soluble (chemotactic) and bound (haptotactic) chemokine gradients were obtained. The media in the upper chamber were removed and replaced with 2 × 10^5^ resting, naïve, memory or activated CD4+ T cells in 100 μL of 10% AB serum in RPMI (Gibco). Following one hour incubation with HBMEC at 37 °C in a 5% CO2/95% air incubator, the non-adherent cells were removed by gently pipetting up and down at each of the lateral walls of the upper chamber. The HBMEC along with the adherent leukocytes were fixed with ice cold 1:1 acetone/ethanol for 7 min at 4 °C. Mouse anti-human leukocyte common antigen (LCA/CD45) and horseradish peroxidase (HRP)-conjugated goat anti-mouse IgG were used for staining adherent lymphocytes in adhesion experiments. Light counter-staining with hematoxylin allowed visualization of the nuclei and perinuclear cytoplasm of HBMEC. The number of adherent T cells was determined by counting 1 central and 8 peripheral fields using a 20× objective lens and a 1-cm^2^ grid as previously described [56]. All counts were performed blindly. Results are expressed as the number of adherent leukocytes per square millimeter of the HBMEC monolayer.

### 3.8. Statistics

Statistical analyses were performed using GraphPad Prism 4.03 (GraphPad Software, San Diego, CA, USA). Student’s *t*-test was used to compare means between two groups. For three or more groups, one way analysis of variance (ANOVA) was performed to determine differences between treatment groups, followed by Dunnett’s post test to analyze individual differences against control groups. Non-normally distributed data was analyzed by Mann-Whitney test or Kruskal-Wallis test followed by Dunn’s multiple comparison test. *p* values less than 0.05 were considered statistically significant.

## 4. Conclusions

Perivascular infiltrates of lymphocytes and monocytes recruited to the brain across the normally restrictive BBB are a characteristic histopathologic feature of the MS lesion. Chemokines play a central role in the recruitment of leukocytes to inflammatory sites, however, due to the complexity of the chemokine family and the intricate interactions with their receptors, the function of the various chemokines in attracting and facilitating the entry of specific types of leukocytes into the brain has not been fully investigated. The results of the present study support a role for the β-chemokines CCL2 and CCl3 in the preferential adhesion of CD4+ T cell subsets to resting and activated human brain endothelial cells. Recently activated T cells responded to CCL2 by increased adhesion to cytokine-activated, but not resting, endothelial cells. CCL3 specifically supported the adhesion of memory T cells to both resting and stimulated endothelium. The differential effects of the various chemokines on the adhesion and homing of different lymphocyte subsets to the CNS is likely an important factor in defining the composition of the inflammatory infiltrates at different stages of the MS lesions and other inflammatory CNS diseases.
